# Supplementary material for: Multiple capsid protein binding sites mediate selective packaging of the alphavirus genomic RNA
Source: Nat Commun. 2020 Sep 17;11:4693. doi: 10.1038/s41467-020-18447-z (PMC7499256; doi:10.1038/s41467-020-18447-z)
Supplement: Supplementary file 1 — Supplementary Information [file 41467_2020_18447_MOESM1_ESM.pdf]

SUPPLEMENTARY INFORMATION

MULTIPLE CAPSID PROTEIN BINDING SITES MEDIATE SELECTIVE PACKAGING OF THE ALPHAVIRUS GENOMIC RNA

Brown et al.

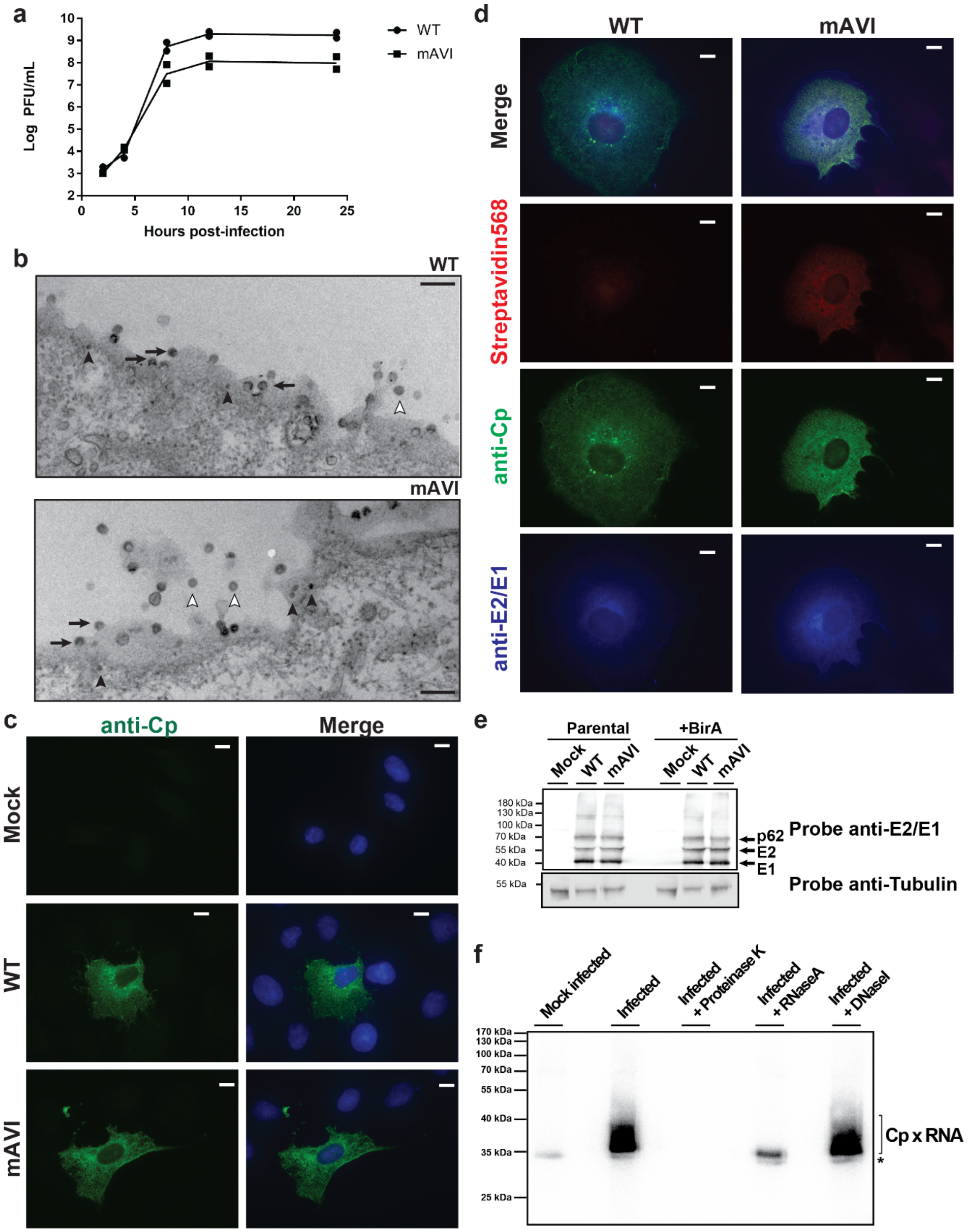

Supplementary Figure 1. SFV/Cp mAVI virus is comparable to SFV/Cp WT. Related to Figure 1. (a) Single-step growth curve comparing SFV WT vs. mAVI. Cells were infected at an MOI=10 and the media collected at the indicated time points and titered by plaque assay. Individual points from n=2 biological replicates are plotted. Source data are provided as a Source Data file. (b) Transmission electron microscopy

of Vero BirA cells infected for 7.5 h with SFV WT (upper) or SFV mAVI (lower). Scale bar represents 200 nm. Black arrows denote NCs of budding viral particles, white arrowheads denote budded viral particles, and black arrowheads denote cellular NCs. The images are representative examples from n=1 experiment. (c) Immunofluorescence of Vero cells mock infected or infected with SFV WT or SFV mAVI for 7.5 h and stained with a monoclonal antibody against Cp. Scale bar represents 10 µm. The images are representative examples from n=2 biological replicates. (d) Immunofluorescence of Vero BirA cells infected with SFV WT or SFV mAVI for 7.5 h and stained with a monoclonal antibody against Cp (green), a polyclonal antibody against E2/E1 (blue), and a Streptavidin Alexa-568 probe (red). Scale bar represents 10 µm. The images are representative examples from n=2 biological replicates. (e) The lysates from Figure 1B were analyzed by western blot using a polyclonal antibody to envelope proteins E2/E1 and a polyclonal antibody to tubulin. The images are representative examples from n=2 biological replicates. Source data are provided as a Source Data file. (f) PAR-CLIP was performed on Vero BirA cells mock infected or infected with SFV mAVI. SA-DB-retrieved samples were ^{32}P-end labeled and either mock treated or treated with Proteinase K, RNaseA, or DNaseI to identify the nature of the lower band (asterisk). The lower band is a UV-independent (Figure 1e) and proteinaceous species. The image represents n=1 experiment. Source data are provided as a Source Data file.

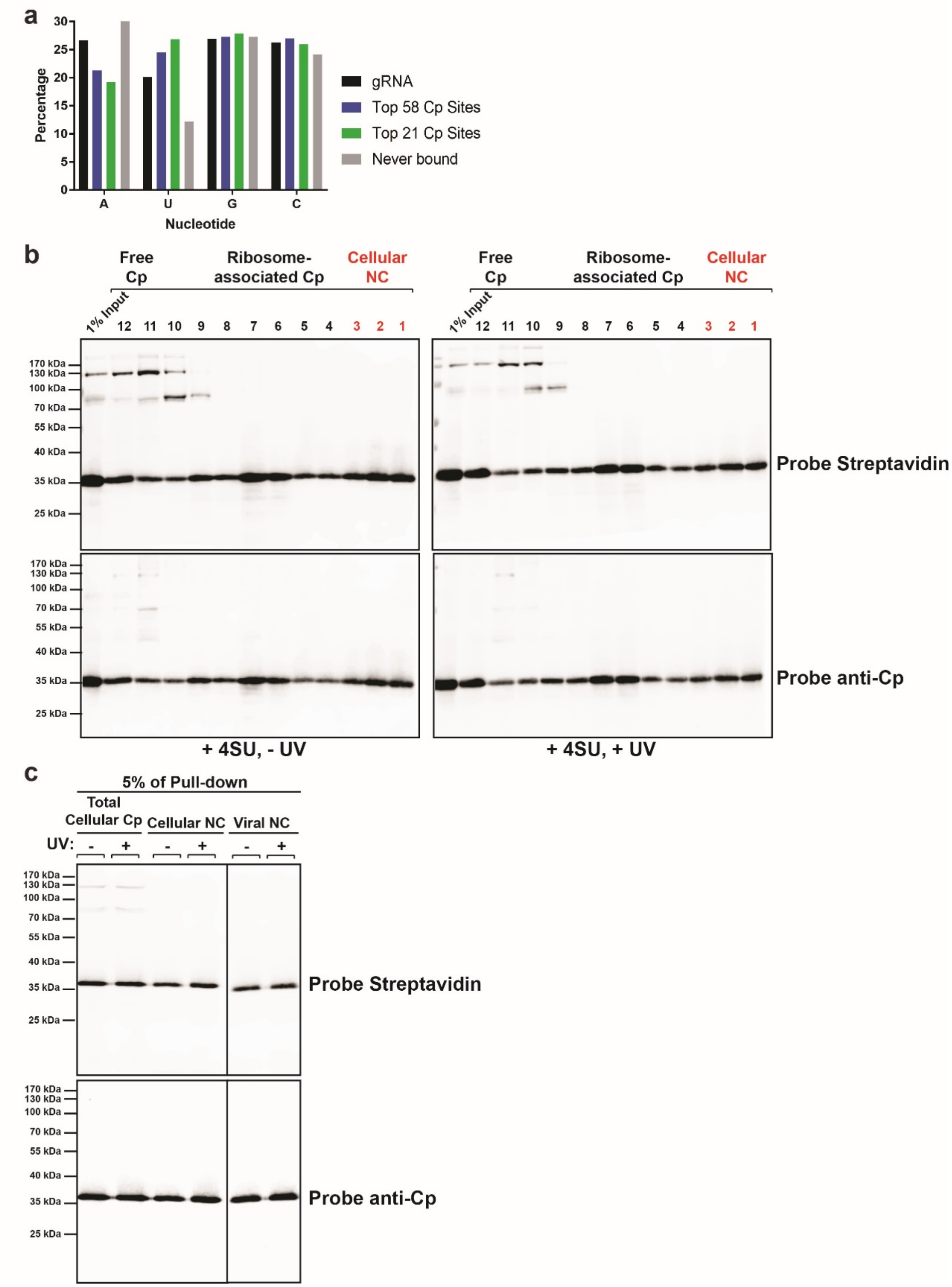

Supplementary Figure 2. PAR-CLIP sample preparation. Related to Figures 2 and 5. (a) Nucleotide composition of the genome (gRNA), the top 58 Cp binding sites, the top 21 Cp binding sites, and the sites never bound by Cp (zero reads). (b) Vero cells were infected at an MOI=10 with SFV mAVI and incubated with 4SU. At 7 hpi, cells

were UV irradiated or mock irradiated, lysed, processed, and the cellular NCs were fractionated by 7.5-20% (wt/wt) sucrose gradient sedimentation. Aliquots of each fraction were subjected to SDS-PAGE and western blot analysis using a polyclonal antibody to Cp and a Streptavidin Alexa-680 probe. Cellular NC fractions (red) were pooled, denatured with 0.5% SDS, and then incubated with SA-DB to isolate CpxRNA complexes. The images are representative examples from n=2 biological replicates. Source data are provided as a Source Data file. (c) Cp PAR-CLIP libraries from the total cellular, cellular NC, and viral NC populations were generated using similar starting protein concentrations. 5% of each of the SA-DB-retrieved samples was analyzed by western blot using a monoclonal antibody to Cp and a Streptavidin Alexa-680 probe. Samples are from the same gel/blot, but the lanes were rearranged post-image acquisition for lane consistency and therefore a black line was added between cellular and viral NC. The images represent n=2 independent experiments. Source data are provided as a Source Data file.

a GLAM2 motif analysis of top 21 and top 58 binding sites.

Cp's top 21 sites. Best Motif Found:

NAME	START	SITES	END	STRAND	MARGINAL SCORE
1	11	cgg.gtcg	17	+	1.53
2	16	tggagttg	23	+	8.50
3	16	tggtgttg	23	+	8.87
4	5	tgctgttc	12	+	6.53
5	9	ggcaactc	16	+	3.38
6	25	tggccttg	32	+	6.26
7	10	gggagcgg	17	+	1.65
8	11	tgg.attg	17	+	5.53
9	11	ggccgctg	18	+	7.67
10	11	tgc.gttt	17	+	1.30
11	10	tgctgctg	17	+	7.96
12	15	tgctattg	22	+	7.51
13	5	ggccgtcg	12	+	6.23
14	5	tggagttg	12	+	8.50
15	23	ggg.gccg	29	+	2.97
16	37	tgacactg	44	+	4.72
17	3	tgccgtcc	10	+	4.89
18	24	ggg.attc	30	+	2.52
19	11	cgacgttg	18	+	3.64
20	7	gggtactg	14	+	6.09
21	14	tgccccctg	21	+	5.35

Score: **81.8401**

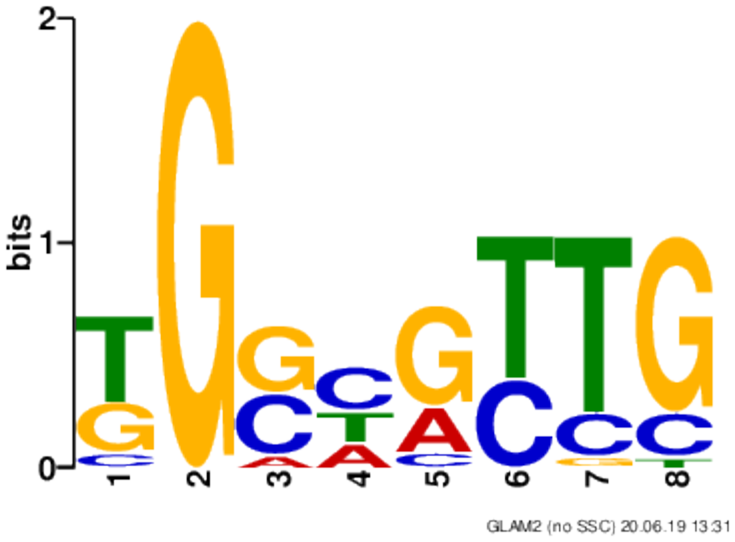

Cp's top 58 sites. Best Motif Found:

NAME	START	SITES	END	STRAND	MARGINAL SCORE
1	4	tgg.ttg.acggg.t.cg	17	+	3.01
2	10	cgg.ctg.tggag.t.tg	23	+	12.2
3	4	aag.cg..tcgggtt.tg	17	+	5.58
4	8	tgttct..ccaactt.tg	22	+	6.36
6	19	cag.tgg.tggcc.t.tg	32	+	5.38
7	3	tgt.ac..acgggag.cg	16	+	1.21
8	11	tgg.at..t.gggtt.tg	23	+	4.23
9	23	cgg.cc..tggac.cccg	36	+	7.41
11	5	tta.ct..tgctg.c.tg	17	+	1.27
13	4	agg.ccg.tcggcac.ca	18	+	3.67
14	11	tga.cc..tggacag.tg	24	+	10.4
15	16	agg.ct..tggggc.cg	29	+	5.21
16	32	cgt.ct..t.gacac.tg	44	+	8.73
17	6	cgtccgg.tcatctt.tg	21	+	4.56
18	3	cgc.cg..t.gac.g.ta	14	+	1.13
19	5	cga.cg..tcgacgt.tg	18	+	11.2
20	29	tga.ct..c.cgc.c.tg	40	+	2.47
21	22	cgt.cgg.agcac.g.tg	35	+	7.75
22	7	tga.ct..tggac.t.gg	19	+	4.73
24	15	tgctctg.tacac.t.cg	29	+	5.01
25	5	cga.ttg.tggccgt.cg	19	+	5.45
26	17	cgttcg..tcatc.c.cg	30	+	2.89
28	24	tgt.cc..tggacac.tg	37	+	11.5
31	11	cgt.cgt.tcgag.g.tg	24	+	5.00
32	17	cgg.ctg.c.gcctg.tg	30	+	6.40
34	6	tga.cg..tacac.cctg	19	+	5.01
35	5	tga.ctg.t.atctt.cg	18	+	3.91
36	9	ttg.cg..cccggat.tg	22	+	2.69
38	7	cgg.ccg.aagactt.tg	21	+	9.35
39	2	agttccg.tggacgc.tg	17	+	9.65
40	23	cgc.ccg.tggaa.c.tg	36	+	4.94
41	7	aac.at..aggactg.tg	20	+	0.690
42	41	tgg.agg.ccgag.c.tg	54	+	7.83
43	17	cgc.cg..tacggatctg	31	+	3.01
45	8	cgttcgg.c.gactt.tg	22	+	8.80
46	6	tac.ctgctggac.t.tg	20	+	5.57
48	1	tat.ccg.cggtcgt.cg	15	+	5.54
52	9	tta.cgg.ccgccgg.tg	23	+	4.75
53	15	tgg.ac..tcggcag.tg	28	+	6.94
54	4	tgt.cgg.tggtcacctg	19	+	11.1
55	2	caa.cg..cggac.c.tg	14	+	6.96
57	41	tgg.agg.ccgag.c.tg	54	+	7.83
58	10	cag.cgg.a.gccat.tg	23	+	4.82

Score: **145.701**

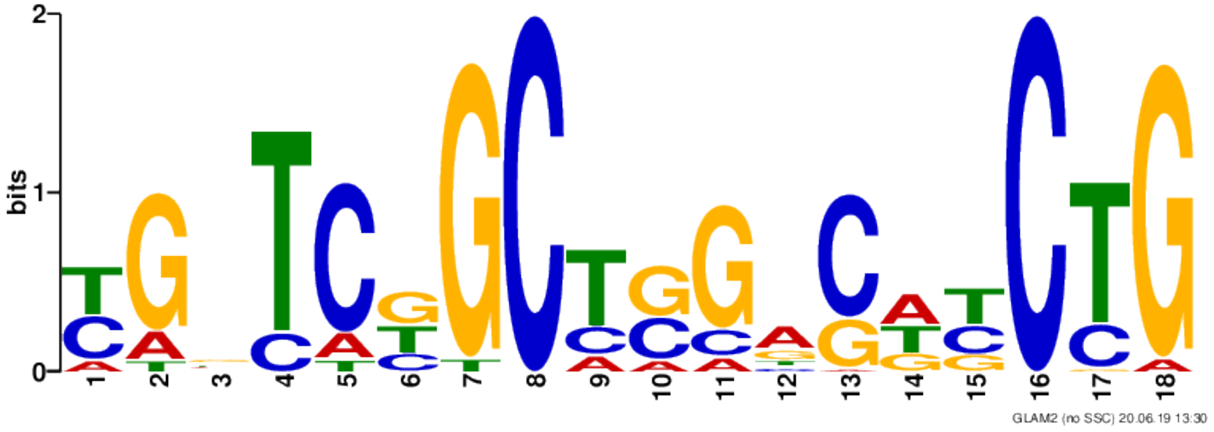

bits
GLAM2 (no SSC) 20.06.19 13:30

b **mFold secondary structure predictions of top 21 binding sites.**

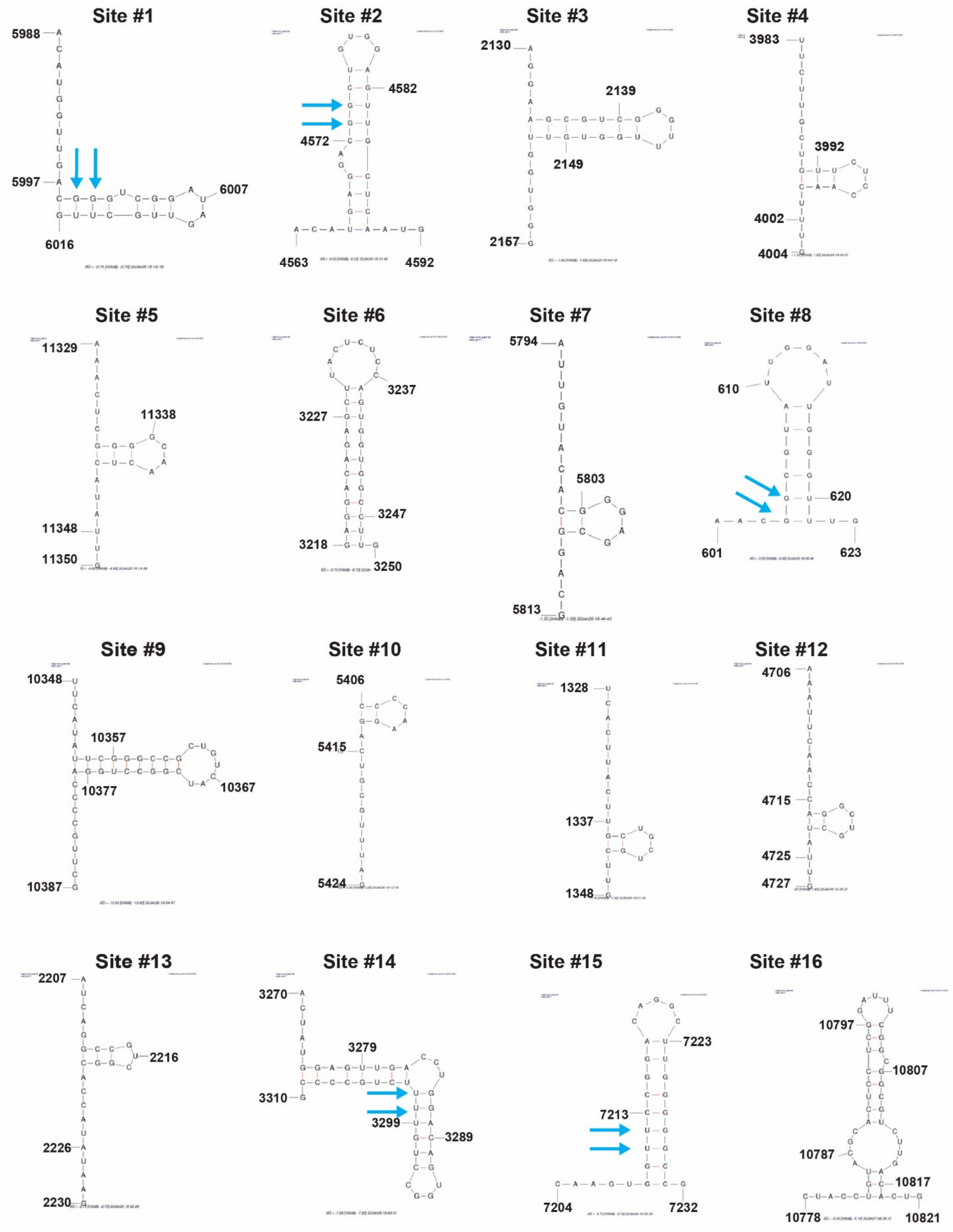

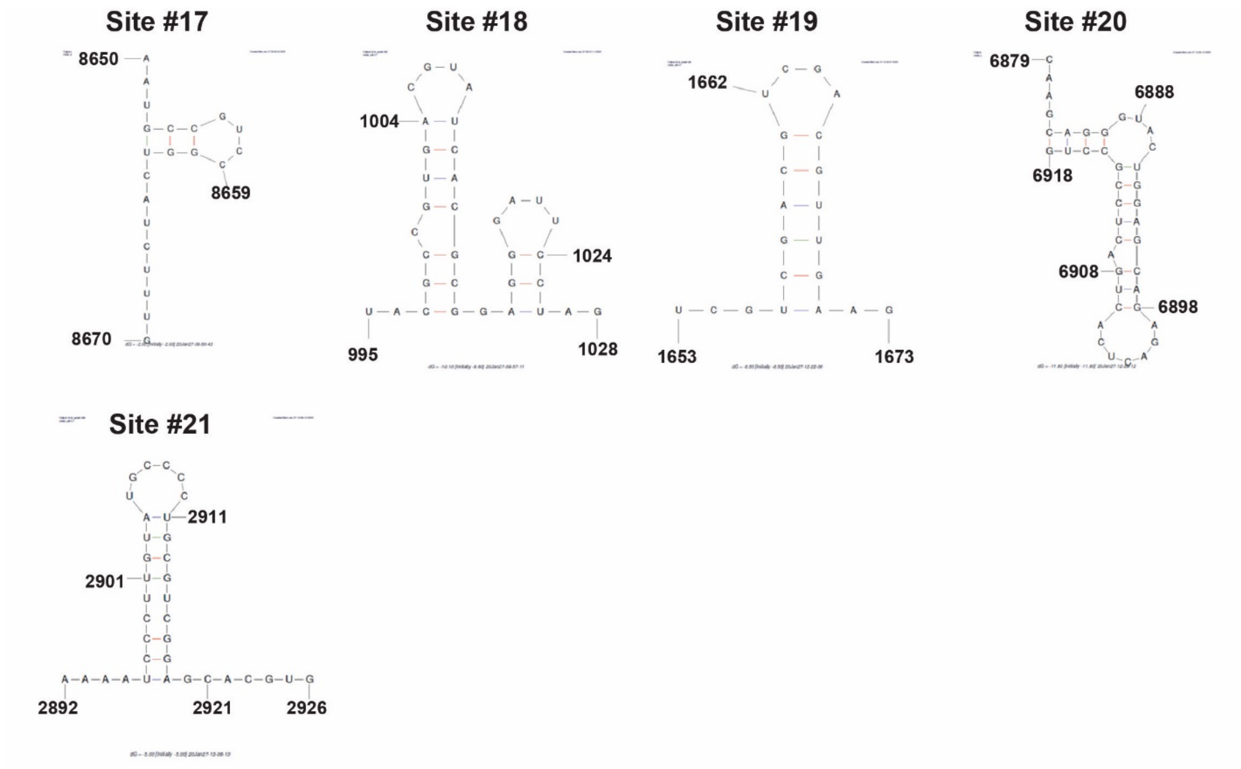

c **Comparison of different mFold secondary structure predictions for site #1.**

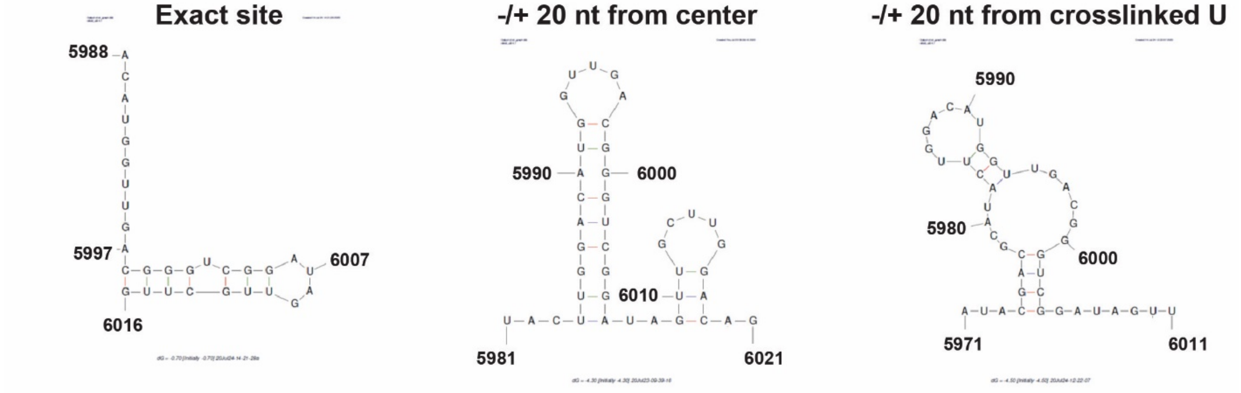

d **Comparison of different mFold secondary structure predictions for site #2.**

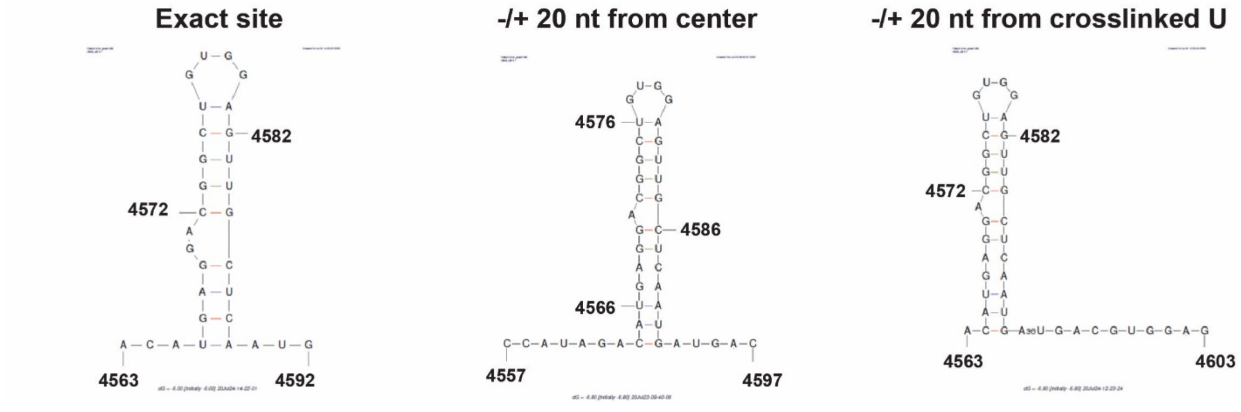

e **mFold secondary structure predictions of SFV PS WT vs. Mutant.**

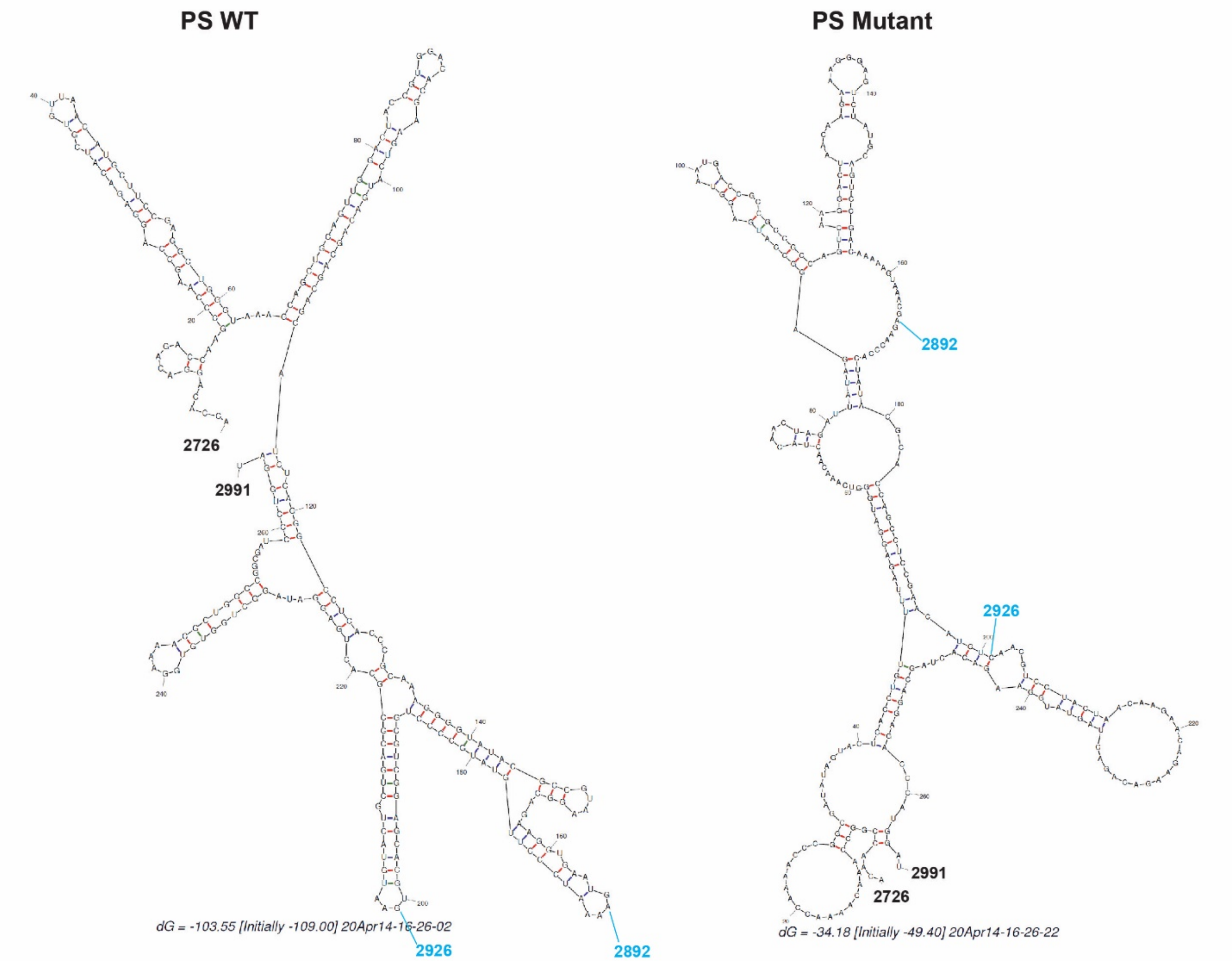

Supplementary Figure 3. Sequence and structural motif analyses for Cp's top binding sites. Related to Figures 2 and 6. (a) GLAM2 was used with its default settings to discover potential gapped sequence motifs within Cp's top 21 and top 58 binding sites. (b) RNA secondary structure predictions for Cp's top 21 binding sites using mFold under default settings. Input sequence was the exact sequence identified as the binding site. Genome nucleotide positions are labeled. Blue arrows indicate tandem G:U wobble base pairs present in sites #1, 2, 8, 14, and 15. (c) mFold secondary structure predictions for site #1 RNA using different input sequences: the exact sequence identified as the binding site (left), -/+20 nt from the center nucleotide within the binding site (middle), and -/+20 nt from the U that crosslinked most frequently (right). (d) As in (c) but for site #2 RNA. (e) RNA secondary structure prediction for the WT PS sequence (nt:2726-2991) (left) compared to the mutated sequence of the Full PS mutant (right). Blue numbers mark the Cp-PS binding site's 5' and 3' ends.

a

Overall sequence identity between SFV and CHIKV_AF15561 is ~67%

```
Site 1
27/29 93%
SFV            ACATGGTTGACGGGTCGGATAGTTGCTTG
AF15561        ACATGGTGGACGGGTCGGAGAGTTGCTTG
               ******* *********** *********

Site 2
21/30 70%
SFV            ACATGAGGACGGCTGTGGAGTTGCTCAATG
AF15561        AGATGCGGACCCAAGTGGAGCTGCTGGATG
               * *** ****    ******.**** .***

Site 3
20/28 71%
SFV            AGGAAGCGTCGGGTTTGGTGTTGGTGGG
AF15561        AAGAAGCTGCAGGACTGGTACTGGTGGG
               *.*****  *.** .****..*******

Site 4
15/22 68%
SFV            TTCTTGCTGTTCTCCAACTTTG
AF15561        TTTTTCCTATTTAGCAATTTTG
               **.** **.**.  ***.****

Site 5
12/22 54.5%
SFV            AAAC-TCGGGGCAACTCATATTG
AF15561        GGACGTAGGAGATGTTCAAAGTG
               ..** * **.*  ..*** * **

Site 6
23/32 72%
SFV            AGGACAGAGCTTACTCTCCAGTGGTGGCCTTG
AF15561        AAGACAAAGCATACTCACCCGAAGTAGCCCTG
               *.****.*** ***** ** * .**.***.**

Site 7
11/20 55%
SFV            ATTGTACACGGGAGCGGA-----CG
AF15561        ACTGTATTTAATGGCAGAGACCCCG
               *.****. ... .**.**     **

Site 8
17/23 74%
SFV            AACGGCGTATTGGATTGGGTTTG
AF15561        AGTGGCGTACTGGGTAGGGTTCG
               *..******.***.* *****.*

Site 9
30/40 75%
SFV            TTCATATTCGGGCCGCTGTCATCGGCCTGGACCCCGTTCG
AF15561        TTCATTGTGGGGCCAATGTCTTCAGCCTGGACACCTTTTG
               *****  * *****..**** **.******** ** **.*
```

```
Site 10
11/19 58%
SFV           CC------------------------CCAAGGACT------------------------
AF15561       ACACAGCTATTTCCCTTCAGGCACCGCCAAGTACCACCATGGAACTGAGCCATCCACCG
               *                        ***** **.
SFV           ------------GCGTTTAG
AF15561       ATCTCCTTCGGAGCACCAAG
                          * ... **

Site 11
15/21 71%
SFV           TCACTTACTTGCTGCTGCTTG
AF15561       ACACTAACCTGCTGCTGTCTA
               **** **.********..*.

Site 12
12/22 55%
SFV           AAATTCAACCAGGCTGCTATTG
af15561       CGTTTTCACCAGACGGCAGTGG
               . **. *****.* ** .* *

Site 13
16/24 67%
SFV           ATCAG-GCCGTCGGCACCATATAAG
AF15561       ATTCGCCCCGCCTGC-CCATACAAA
              **. *  ***.* ** *****.**.

Site 14
30/42 71%
SFV           ACTATGGAGTTGACCTGGACAGTGGCCTGTTTTCTGCCCCG
AF15561       TGTATGGGGTGGATCTAGACAGTGGGCTATTCTCTAAACCG
                *****.** **.**.******** **.**.***.  ***

Site 15
16/29 55%
SFV           CAAGTGGTTCCGGACAGGCTTGGGGGCCGA
AF15561       CAGATGGCAACGAACAGGGCTAATAGATGA
              **..***.  **.***** .*.. .* .**

Site 16
28/44 66%
SFV           CTACCTGTACGCACTCCTCGGATTTCGGCGGCGTCTTGACACTG
AF15561       CAGCCTGCACCCACTCCTCAGACTTTGGGGGCGTAGCCATCATT
              * .****.** ********.**.**.** *****    *.  *

Site 17
11/21 52%
SFV           AATGCCGTCCGGTCATCTTTG
AF15561       AGGGCCG---GGCTATTTGT-
              *. ****   **..**.* *

Site 18
24/34 71%
SFV           TACGCCGTGACGTATCACGCGGAGGGATTCCTAG
AF15561       TATGCGGTAACCCACCACGCAGACGGATTCTTGA
              **.** **.** .*.*****.** ******.*..
```

```
Site 19
17/21 81%
SFV            TCGACGTCGACGTTGAAG
AF15561        TCGAAATCGACGTGGAAC
               **** .******* ***
```

```
Site 20
26/40 65%
SFV            CAAGCAGGGTACTGGAGCAGAGACTCACTGACTCCGCCTG
AF15561        CTAGCCGGGTGTTGGAAGATCGTCTGACAAAATCCGCATG
               * *** ****..****. *  * ** ** .* ***** **
```

```
Packaging Sequence Binding Site
Site 21
24/35 69%
SFV            AAAATCCCTTGTATGCCCCTGCGTCGGAGCACGTG
AF15561        AAAACCCACTCTATGCATCAACATCAGAGCACGTC
               ****.** .* ***** .* .*.**.********
```

b

```
Full Packaging Sequence
197/266 74%

SFV            ACCACAGGACAGACCAAGCCCAAGCCAGGAGACATCGTGTTAACATGCTTCCGAGGCTGGG
AF15561        ACTACAGGCTCAACGAAACCTGACCCTGGAGACCTCGTGTTAACGTGCTTCAGAGGGTGGG
               **.***** . .** **.**..* ** ****** **********.****** **** ****

SFV            TAAAGCAGCTGCAGTTGGACTACCGTGGACACGAAGTCATGACAGCAGCAGCATCTCAGGG
AF15561        TTAAACAACTGCAAATTGACTATCGTGGACACGAGGTCATGACAGCAGCCGCATCCCAAGG
               * **.**.*****. * *****.***********.************** *****.**.**

SFV            CCTCACCCGCAAAGGGGTATACGCCGTAAGGCAGAAGGTGAATGAAAATCCCTTGTATGCC
AF15561        GTTAACTAGAAAAGGAGTTTACGCAGTTAGGCAAAAAGTTAACGAAAACCCACTCTATGCA
                .* **. * *****.** ***** ** *****.**.** **.*****.** .* *****

SFV            CCTGCGTCGGAGCACGTGAATGTACTGCTGACGCGCACTGAGGATAGGCTGGTGTGGAAAA
AF15561        TCAACATCAGAGCACGTCAACGTACTCCTAACGCGTACGGAAGGTAAACTGGTATGGAAGA
               .* .*.**.******** **.***** **.*****.** **.*.**..*****.*****.*

SFV            CGCTGGCCGGCGATCCCTGGAT
AF15561        CACTCTCTGGTGACCCGTGGAT
               *.**  *.**.**.** *****
```

Supplementary Figure 4. Sequence alignments between SFV and CHIKV. Related to Figures 2 and 4. Multiple sequence alignment (MUSCLE)[1] was used to align the SFV and CHIKV genome sequences. (a) The top 21 SFV Cp binding sites aligned with the corresponding CHIKV sequences. Sequences highlighted in yellow are hyperconserved between SFV and CHIKV compared to the overall sequence identity (~67%) between SFV and CHIKV genomes. (b) The SFV full PS (nt:2726-2991) aligned with the corresponding CHIKV sequence. The sequence is highlighted in yellow because it is hyperconserved.

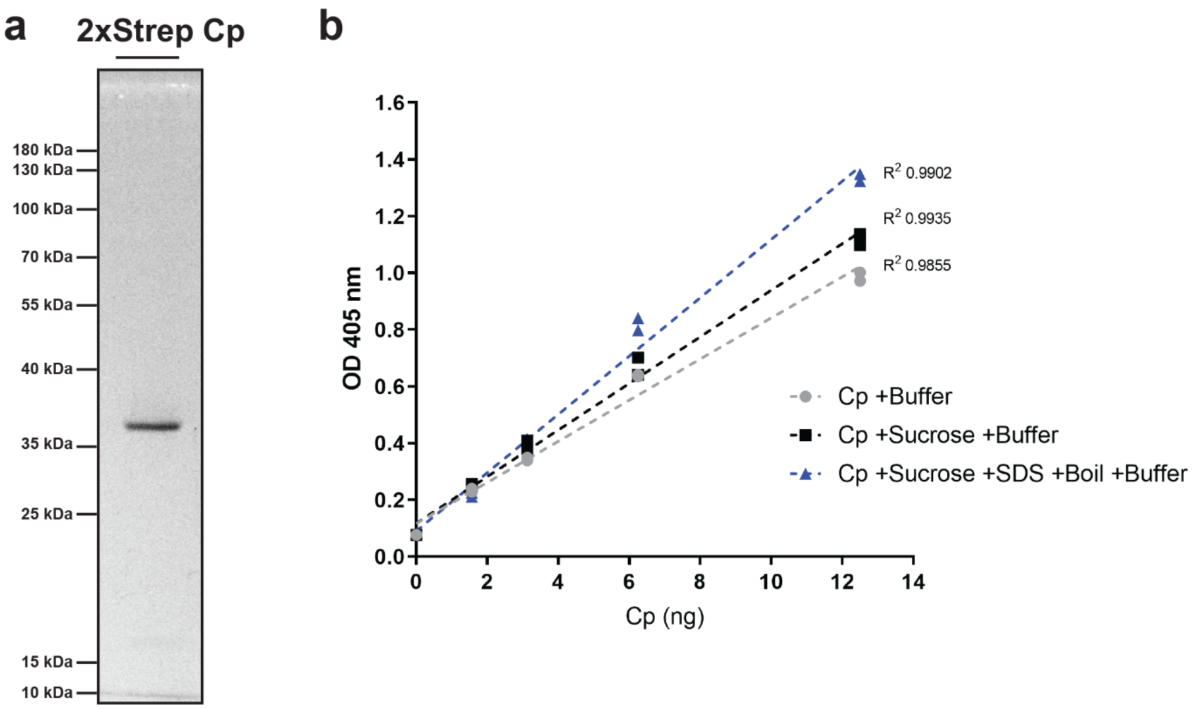

Supplementary Figure 5. Purified Cp and Cp ELISA. Related to Figure 6. (a) 300 ng of purified 2xStrep-tagged Cp was subjected to SDS-PAGE and Coomassie staining. The gel is representative of n=5 independent purifications. Source data are provided as a Source Data file. (b) ELISA signals corresponding to different Cp treatment conditions. A 2-fold dilution series of Cp was incubated either 30% sucrose, or 30% sucrose and 1% SDS plus boiling for 5 min before diluting in binding buffer. Individual absorbance readings from technical duplicates are plotted. Lines represent linear regression. ELISA signals between the various treatments were comparable and linear (R^2>0.98) within the same protein range (~1-12.5 ng). The graph is representative of n=2 independent experiments. Source data are provided as a Source Data file.

Supplementary References

1. Madeira, F. *et al.* The EMBL-EBI search and sequence analysis tools APIs in 2019. *Nucleic Acids Res* **47**, W636-w641 (2019).
