# Supplementary material for: Multiple capsid protein binding sites mediate selective packaging of the alphavirus genomic RNA
Source: Nat Commun. 2020 Sep 17;11:4693. doi: 10.1038/s41467-020-18447-z (PMC7499256; doi:10.1038/s41467-020-18447-z)
Supplement: Supplementary file 2 — Reporting Summary [file 41467_2020_18447_MOESM2_ESM.pdf]

nature research

Corresponding author(s): Margaret Kielian

Last updated by author(s): Aug 17, 2020

Reporting Summary

Nature Research wishes to improve the reproducibility of the work that we publish. This form provides structure for consistency and transparency in reporting. For further information on Nature Research policies, seeAuthors & Referees and theEditorial Policy Checklist .

Statistics

For all statistical analyses, confirm that the following items are present in the figure legend, table legend, main text, or Methods section.

n/a	Confirmed	
☐	☒	The exact sample size (n) for each experimental group/condition, given as a discrete number and unit of measurement
☐	☒	A statement on whether measurements were taken from distinct samples or whether the same sample was measured repeatedly
☐	☒	The statistical test(s) used AND whether they are one- or two-sided<br>*Only common tests should be described solely by name; describe more complex techniques in the Methods section.*
☒	☐	A description of all covariates tested
☐	☒	A description of any assumptions or corrections, such as tests of normality and adjustment for multiple comparisons
☐	☒	A full description of the statistical parameters including central tendency (e.g. means) or other basic estimates (e.g. regression coefficient) AND variation (e.g. standard deviation) or associated estimates of uncertainty (e.g. confidence intervals)
☐	☒	For null hypothesis testing, the test statistic (e.g. F, t, r) with confidence intervals, effect sizes, degrees of freedom and P value noted<br>*Give P values as exact values whenever suitable.*
☒	☐	For Bayesian analysis, information on the choice of priors and Markov chain Monte Carlo settings
☒	☐	For hierarchical and complex designs, identification of the appropriate level for tests and full reporting of outcomes
☐	☒	Estimates of effect sizes (e.g. Cohen's d, Pearson's r), indicating how they were calculated

Our web collection on statistics for biologists contains articles on many of the points above.

Software and code

Policy information about availability of computer code

Data collection	No software was used for data collection.
Data analysis	Adapters were removed using Cutadapt 2.4 (Ref 72: Martin, M. 2011. EMBnet.journal) and the remaining sequences were mapped to the SFV4 genome using Bowtie/1.1.1. Only mapped sequences that contained the characteristic T-to-C mutation were further used. See Methods for details. Additional software used: Mfold 3.6, Graphpad Prism 7.04, Adobe Photoshop CS5.1, and Adobe Illustrator CS5.1.

For manuscripts utilizing custom algorithms or software that are central to the research but not yet described in published literature, software must be made available to editors/reviewers. We strongly encourage code deposition in a community repository (e.g. GitHub). See the Nature Research guidelines for submitting code & software for further information.

Data

Policy information about availability of data

All manuscripts must include a data availability statement. This statement should provide the following information, where applicable:

- Accession codes, unique identifiers, or web links for publicly available datasets
- A list of figures that have associated raw data
- A description of any restrictions on data availability

The PAR-CLIP datasets are publicly available in the NCBI GEO repository under GEO accession number GSE156313 [https://www.ncbi.nlm.nih.gov/geo/query/acc.cgi?acc=GSE156313]. Source data are provided with this paper. The source PDB file used to create the image in Figure 1a is publicly available in the RCSB Protein Data Bank as PDB accession number 6MX7 [https://www.rcsb.org/structure/6MX7].

Field-specific reporting

Please select the one below that is the best fit for your research. If you are not sure, read the appropriate sections before making your selection.

☒ Life sciences ☐ Behavioural & social sciences ☐ Ecological, evolutionary & environmental sciences

For a reference copy of the document with all sections, see nature.com/documents/nr-reporting-summary-flat.pdf

Life sciences study design

All studies must disclose on these points even when the disclosure is negative.

Sample size	We performed n=2 for the PAR-CLIP libraries, which showed excellent correlation. This is the typical sample size for PAR-CLIP libraries.
Data exclusions	No data were excluded.
Replication	All experiments were performed at least twice, details listed in Legends and Methods. The negative stain TEM in Figure 6b was performed once (listed in Figure Legend), but corroborated with sucrose gradient sedimentation analyses in Figure 6c and 6d. The TEM in Supplementary Figure 1b was performed once (listed in Figure Legend), but corroborated with sucrose gradient sedimentation analyses (Supplementary Figure 2b). Sample sizes were chosen based on typical experimental variation and on the ability to confirm results with other assays.
Randomization	No groups were assigned, no randomization was needed.
Blinding	No groups were assigned, no blinding was required.

Reporting for specific materials, systems and methods

We require information from authors about some types of materials, experimental systems and methods used in many studies. Here, indicate whether each material, system or method listed is relevant to your study. If you are not sure if a list item applies to your research, read the appropriate section before selecting a response.

Materials & experimental systems

n/a	Involved in the study	
☐	☒	Antibodies
☐	☒	Eukaryotic cell lines
☒	☐	Palaeontology
☒	☐	Animals and other organisms
☒	☐	Human research participants
☒	☐	Clinical data

Methods

n/a	Involved in the study	
☒	☐	ChIP-seq
☒	☐	Flow cytometry
☒	☐	MRI-based neuroimaging

Antibodies

Antibodies used	Monoclonal anti-β tubulin (E7) (WB-1:3000; Developmental Studies Hybridoma Bank). Rabbit polyclonal anti-α tubulin (WB-1:5000; Abcam, 18251). Rabbit polyclonal anti-E2/E1 (WB-1:4000 and IF-1:500) (Ref 60: Ahn, et al. 1999. JVI). Monoclonal anti-Cp (42-1) (WB-1:4000 and IF-1:500) (Ref 61: Greiser-Wilke, et al. 1989. JGenVirol). Monoclonal anti-E2 (E2-1) (Ref 62: Kielian, et al. 1990. JVI) was used from the clarified hybridoma supernatant at a 1:2 dilution for western blot. Streptavidin Alexa Fluor 680 Conjugate (WB-1:10000; ThermoFisher Scientific, S32358). Streptavidin Alexa Fluor 568 Conjugate (IF-1:700; ThermoFisher Scientific, S11226). Rabbit polyclonal anti-nsP2 (WB-1:3000) was a gift from and was developed by Dr. Andres Merits (University of Tartu, Estonia) (DOI: 10.1074/jbc.M113.503433). Rabbit polyclonal anti-Cp (WB-1:4000, IF-1:500, ELISA-1:1000) was generated by our lab against the C-terminal domain of SFV Cp (residues 119-267), expressed and purified as described in the Methods, and a rabbit was immunized using a commercial vendor (Covance). Secondary antibodies: goat-anti-rabbit IgG AP (ELISA-1:1000; Southern Biotech, 4049-04), goat-anti-rabbit IgG DyLight 800 (WB-1:10000; ThermoFisher Scientific, SA5-35571), goat-anti-mouse IgG Alexa Fluor 680 (WB-1:10000; ThermoFisher Scientific, A-21057), goat-anti-rabbit IgG Alexa Fluor 680 (WB-1:10000; ThermoFisher Scientific, A-21076), goat-anti-mouse IgG Alexa Fluor Plus 800 (WB-1:10000; ThermoFisher Scientific, A32730), goat-anti-rabbit IgG Alexa Fluor 405 (IF-1:700; ThermoFisher Scientific, A-31556), goat-anti-mouse IgG Alexa Fluor 488 (IF-1:700; ThermoFisher Scientific, A1-11001).
Validation	See above. Antibodies have been extensively validated for recognition of Semliki Forest virus E2, E1, capsid, and nsP2 proteins by immunofluorescence and western blot analyses of uninfected vs. infected cells. The capsid protein rabbit serum was tested for recognition of SFV Cp by western blot and immunofluorescence of uninfected vs. infected cells, and co-staining with the monoclonal anti-Cp antibody (42-1). Commercial antibodies were validated by their specific source vendors.

Eukaryotic cell lines

Policy information about cell lines

Cell line source(s)	Baby hamster kidney (BHK-21) cells were a gift from Dr. Ari Helenius, and were originally obtained from ATCC. Vero cells were from ATCC and obtained from Dr. Kartik Chandran (Albert Einstein College of Medicine).
Authentication	Morphological.
Mycoplasma contamination	All cells were tested for mycoplasma contamination using the MycoAlertTM PLUS Mycoplasma Detection Kit from Lonza and were negative.
Commonly misidentified lines (See ICLAC register)	None.
