# Supplementary material for: Multiple capsid protein binding sites mediate selective packaging of the alphavirus genomic RNA
Source: Nat Commun. 2020 Sep 17;11:4693. doi: 10.1038/s41467-020-18447-z (PMC7499256; doi:10.1038/s41467-020-18447-z)
Supplement: Supplementary file 3 — Description of Additional Supplementary Files [file 41467_2020_18447_MOESM3_ESM.pdf]

Description of Additional Supplementary Files

File Name: Supplementary Data 1
Description: **Top Cp binding sites.** Specific sequences and average percentile rank of Cp's top binding sites in the total cellular Cp population.

File Name: Supplementary Data 2
Description: **PAR-CLIP libraries.** Summary statistics, reads, and T-to-C frequencies for all PAR-CLIP libraries.

File Name: Supplementary Data 3
Description: **Mutant sequences.** Specific sequences for the 17-site mutant, the SFV PS mutant, and the CHIKV PS mutant.
